# Infinitesimalism Versus Science

**Published:** 1883-07

**Authors:** C. S. Carr


### INFINITESIMALISM VERSUS SCIENCE.

C. S. CARR, M. D.

There are really two schools of homœopaths—high dilutionists and low dilutionists. The low dilutionists, as a class, rarely use remedies above the third or sixth dilution, while the high dilutionists use most, if not all, of their remedies as high as the 30th, 50th, 200th 500th and even 5000th dilutions.

To make the first dilution, one drop of the strong tincture of any drug is taken and added to 99 drops of pure alcohol and thoroughly shaken. Or, in other words, the molecules constituting the drop of the drug used, are diffused through one hundred times as much space as they occupied while in the mother tincture, and the interstices between the molecules are one hundred times as great.

To make the second dilution, one drop of the first dilution is taken and added to 99 drops of pure alcohol and thoroughly shaken, which diffuses the molecules of the original drop through one hundred times as much space as in the first dilution, and ten thousand times as much space as in the original tincture. Or, in other words, the interstices between the molecules of the drug in the second dilution, are ten thousand times as great as in the original tincture.

A molecule is the least possible mechanical division of any matter. To divide a molecule, is to decompose the substance. The identity of any substance depends upon the preservation of its constituent molecules. Hence, we speak of molecules as entities diffused in space, indestructible except by decomposition.

In solid substances we have the molecules in extremely close proximity to each other, but never touching. In fluid substances, the molecules are further apart, and in gasses, still farther. Take, for instance, water; as a fluid we have the molecules composing it very near to each other. But change it to gas by the action of heat and we have the molecules separated or diffused through 1700 times as much space as when they were in the fluid state.

The menstruum in this case being the all prevading ether instead of alchohol; but the physical relation of the molecules being precisely the same. Let us take aconite, for instance, and change it to what is known as the second dilution of that drug. As we have shown, the molecules constituting the drug are diffused through ten thousand times as much space as originally occupied by the drug, while in the case of changing the water to gas, we only have the molecules diffused through 1700 times as much space.

For the sake of obtaining a starting point, we will allow that the molecular separation of aconite in the second dilution is no greater than the molecular separation of water as gas, which is allowing, in favor of high potency, the difference between 1,700 and 10,000.

Here permit me to quote two chemical laws which underlie the new chemistry, viz:

1. "Equal volumes of all substances, when in the state of gas, and under like conditions, contain the same number of molecules."

2. The number of molecules in a cubic inch of any gas at a temperature of 32° F. with an atmospheric pressure as indicated by the barometer at 30 inches is $10^{23}$, or 10 raised to the 23d power, which is 1,000,000,000,000,000,000,000,000, or one septillion.

Now, suppose we have before us a bottle holding a cubic inch of the second dilution of aconite, and another a cubic inch of steam. In the case of the steam we have one septillion molecules as we have shown, while the interstices between the molecules have been widened only 1,700 times; while in the case of the aconite molecules we have widened the interstices ten thousand times.

We know if we had changed our strong tincture to a gas, we should have in our cubic inch one septillion molecules; but we have diffused the molecules in making our second dilution through nearly six times as much space as we would have done had we changed it to a gas.

Allowing for aconite the same diffusion or molecular separation that water has when raised to a gas, we would have in the second dilution of aconite the interstices between the molecules of the aconite nearly six times as great as if the aconite had been raised to a gas. Hence a cubic inch of the second dilution would contain only one-sixth as many molecules as a cubic inch of aconite raised to a gas at the temperature and pressure above named. But we will suppose it to contain just as many. Then we have in a cubic inch of the second dilution of aconite one septillion aconite molecules diffused among alchohol molecules. Now, raise a cubic inch of the second dilution of aconite to the third dilution and the interstices between the aconite molecules are 100 times as great. Or, in other words, one cubic inch would contain 100 times less molecules, or ten sextillion, indicated by $10^{21}$.

Take a cubic inch of the third dilution and raise it to the fourth dilution, which is adding 99 cubic inches of pure alchohol, and we have in any one cubic inch one hundred times less molecules than in any one cubic inch of the third dilution, or the number of molecules is $10^{19}$. So with each additional dilution we drop two of the ciphers which represent the number of molecules contained in each cubic inch. With the 5th dilution we have 17 ciphers or $10^{17}$. In the 6th dilution we have 15 ciphers. In the 7th dilution we have 13 ciphers; in the 8th, 11; in the 9th, 9; in the 10th, 7; in the 11th, 5; in the 12th, 3; in the 13th, 1 or 10 raised to the first power, or 10. Therefore in the 13th dilution we have just 10 molecules to the cubic inch.

Now, let us raise the 13th dilution to the 14th and what do we have? Just one molecule to every ten cubic inches of the dilution; and, in selecting at random any one cubic inch of this 14th dilution we would have one chance in ten of getting a single medicinal molecule. In the 15th dilution, we would run one chance in a thousand of getting a molecule. Then, if I have on my shelf 1,000 vials, each containing a cubic inch of the 15th dilution of any drug, by the law of mathematical probabilities there should be only one molecule in 1,000 vials. Hence only one vial could possibly contain *any* of the original drug and that vial must contain *only one molecule!*

Let us suppose we are prescribing for a patient whose disease demands the administration of a certain drug. Let us grant, for argument sake, that the drug's alleged increased potency, together with the patient's alleged heightened susceptibility to its influence, render one single molecule of the drug sufficient to cure the disease, the patient runs one chance in 1,000 of receiving that molecule by taking a whole cubic inch of the 15th dilution.

The usual method of administering such prepartions is to drop only a few drops of the dilution in a glass of water, of which only a small portion is taken at a dose, which still further jeopardizes the patient's chance of getting the precious medicinal molecule!

If our calculations be true with the 15th dilution (and we challenge refutation) then what becomes of our 30th potency? For it is mathematically demonstrable that there is just one medicinal molecule to one decillion cubic inches of the 30th dilution, or one medicinal molecule to an ocean of alchohol whose bulk is equal to three quintillion nine hundred twenty quadrillion cubic miles. But our whole earth contains only two hundred and sixty-seven billions nine hundred and fifty millions cubic miles.

Hence with the 30th dilution we have one molecule to a bulk of alchohol equal in size to fourteen million globes like ours. Yet we have only crossed the thresheld of our calculations. Ten drops of this monstrous ocean of alchohol in which swims the one infinitessimal molecule, are dropped into a glass of water from which the patient's doses are administered. We have yet to calculate the mathematical probabilities that the precious molecule shall be contained in the tea-spoonful of this potent remedy. But we refrain. Our notation is incompetent to the task.

Then what height of absurdity is reached when rational beings subscribe to the potency of the 50th, 500th, etc., dilution.

The same figures applied to dilutions apply equally to triturations.

The absurdity of infinitesimalism by the decimal scale appears not so soon to be sure, but just as surely and long before the 30th dilution has been reached.

In conclusion, I affirm that there is a limit to the divisibility of any substance without destroying its identity, and that limit is reached when we have made the 13th centesimal dilution.

---

CREAM MEAD.—A very agreeable drink may be prepared for convalescents as follows: Dissolve 3 lbs. of white sugar in ½ gallon of boiling water, and while cold add 3 oz. of tartaric acid previously dissolved in a pint of cold water. Now add the whites of three eggs well beaten, flavor to taste, and bottle. When it is to be used stir in a few grains of bicarbonate of soda, and a delicious effervescing drink is the result.—*Medical Bulletin.*

---

Have you paid your subscription to the BISTOURY for this year? If not, why not? Let us hear from the delinquents at once.

